# Gastrointestinal Symptoms After Sport-Related Concussion: Prevalence and Patterns in a Multi-Cohort Analysis

**DOI:** 10.3390/nu18111740

**Published:** 2026-05-29

**Authors:** Emma Finnegan, Ed Daly, Katherine J. Hunzinger, Lisa Ryan

**Affiliations:** 1 Department of Sport, Exercise and Nutrition, Atlantic Technological University (ATU), Dublin Rd., H91 T8NW Galway, Ireland; emma.finnegan@research.atu.ie (E.F.); ed.daly@atu.ie (E.D.); 2 Department of Exercise Science, Thomas Jefferson University, Philadelphia, PA 19144, USA; katherine.hunzinger@jefferson.edu; 3 Jefferson Center for Injury Research & Prevention, Thomas Jefferson University, Philadelphia, PA 19144, USA

**Keywords:** sport-related concussion, mild traumatic brain injury, gastrointestinal symptoms, athletes, sex differences, symptom assessment, SCAT6, nutrition support

## Abstract

**Background/Objectives:** Concussions occur across all sports globally; however, inconsistent symptom recognition continues to challenge diagnosis, management, and recovery. Although concussion effects extend beyond neurological dysfunction, gastrointestinal (GI) symptoms remain insufficiently captured within current assessment frameworks and may influence athletes’ fuelling choices and food tolerance during recovery. This study aimed to describe the prevalence and patterns of GI symptoms reported among athletes with a history of concussion and to explore whether symptom prevalence and burden differed by sex and country. **Methods**: A total of 401 adult athletes (225 males; mean age 32.4 ± 11.3 years) with a history of concussion were recruited from a multinational cohort (*n* = 123) and a US community rugby cohort (*n* = 278). Participants completed online surveys assessing concussion history, post-concussion symptoms (RPQ or SCAT-6), and GI-specific symptoms. Data were stratified by sex and country and analysed descriptively and comparatively. **Results:** Overall, 62.1% of athletes reported ≥1 GI symptom, with most reporting 1–5 symptoms (79.9%). The most frequent symptoms were nausea/vomiting (74.3%) and loss of/poor appetite (56.6%), followed by diarrhoea, abdominal pain/discomfort, and flatulence. Although GI symptom prevalence was higher in females (65.9% vs. 59.1%), no significant sex differences were observed (all *p* > 0.05). GI symptom burden varied by country (*p* < 0.001), with higher prevalence among Irish than US athletes (85.9% vs. 51.2%); however, these analyses were exploratory and unadjusted. RPQ and SCAT captured symptoms across somatic, cognitive, emotional, and sleep domains, however GI symptoms were underrepresented, with nausea/vomiting more frequently reported on RPQ/SCAT items (51.4%) than on GI-specific items (46.1%). **Conclusions:** GI symptoms were common following concussion, with variation by country and no statistically significant differences by sex. Findings indicate that concussion assessment tools (RPQ/SCAT) underrepresent the breadth of GI symptoms. Greater attention to GI assessment in concussion care is warranted. Incorporating simple GI screening alongside timely nutrition support may represent feasible additions to multidisciplinary, athlete-centred care pathways. Prospective studies are needed to clarify the clinical relevance of these findings and evaluate nutrition-related strategies.

## 1. Introduction

Concussion, a form of mild traumatic brain injury (mTBI), is typically defined by a Glasgow Coma Scale (GCS) score of 13–15 and results from a direct head impact or an impulsive force transmitted through the body to the head [1]. Globally, concussion/mTBI affects an estimated 42–56 million people annually [2,3], with approximately 20–30% occurring in sport and recreational activities [4]. Although sport participation confers substantial physical and psychosocial health benefits [5], athletes in contact, collision, and combat sports face elevated concussion risk due to repeated high-velocity impacts [6,7] and sporting environments that may normalise playing through injury [8].

Concussion diagnosis remains challenging because it relies on athletes’ self-reported symptoms, which are subjective and often difficult to observe [9], and lacks objective diagnostic tests or validated biomarkers [10], contributing to persistent underrecognition in sport [11]. Many concussions go unreported due to limited symptom literacy, misinterpretation of “head knocks” or “bell-ringers” as minor, non-disclosure, and cultural pressures to continue playing [8,11,12]. Consequently, symptoms may be underrecognised or poorly documented, limiting accurate characterisation of concussion-related symptom patterns, particularly those outside core neurological and cognitive domains, such as gastrointestinal (GI) symptoms that are not routinely captured in standard clinical or research frameworks [1,13,14].

Concussion reflects multi-system dysregulation, with symptoms occurring across somatic, cognitive, affective, sleep, and autonomic nervous system domains [15,16] that frequently overlap with conditions such as anxiety and depression, contributing to diagnostic uncertainty [9,16]. Sex-based differences are also reported, with females often experiencing greater somatic and affective symptom severity [17,18]. In sport settings, concussion assessment follows Concussion in Sport Group (CISG) consensus guidelines, with the Sport Concussion Assessment Tool, 6th Edition (SCAT-6) providing multidomain evaluation; however, GI content remains limited, potentially restricting broader symptom recognition [1,10,14].

At a physiological level, concussion results from biomechanical force that causes rapid brain movement and stretching of axonal fibres (strain/deformation) [1,19], initiating an acute neurometabolic energy crisis [20]. This is followed by a transient neurometabolic stress response characterised by ionic imbalance, reduced cerebral blood flow, and metabolic and autonomic dysregulation, which may impair function despite normal neuroimaging [16,21]. During this period, increased metabolic demand may alter energy and nutrient utilisation, suggesting a potential association between concussion-related metabolic stress and nutritional considerations during recovery [22].

Building on this, emerging evidence suggests that concussion-related disturbances may extend beyond the brain, with gut–brain axis (GBA) interactions proposed as a conceptual framework for understanding potential GI symptom expression following concussion [23,24,25]. However, evidence supporting these mechanisms in athletic populations remains limited [26]. Accordingly, GI symptoms following concussion should be interpreted cautiously, as they are non-specific and may reflect interacting physiological, psychological, and contextual factors, including affective symptoms, sleep disturbance, and subjective symptom reporting [1,9,27].

Observational studies in athletic populations report post-concussive GI symptoms such as nausea, vomiting, appetite loss, bloating, abdominal discomfort, constipation, and diarrhoea [13,28,29]. Proposed contributors, including autonomic, gut-related, and microbiome processes, remain exploratory, with evidence suggesting alterations in microbial diversity and composition [24,26]. When present, GI symptoms may reduce appetite, food tolerance and hydration behaviours and coincide with changes in fuelling practices and energy availability during early recovery, particularly when metabolic demands may be elevated [13,22]. Contextual factors such as sport environment, country of residence, cultural norms, and healthcare systems may also influence symptom reporting across athlete cohorts [30,31].

Despite increasing recognition of GI involvement, these symptoms remain underrepresented and are seldom incorporated into concussion assessments. The SCAT-6 includes only one GI-specific item (nausea/vomiting), likely under-capturing broader symptom profiles [1,10]. Few GI- or nutrition-focused guidelines exist post-concussion, and evidence-based nutritional protocols remain limited [13,32]. Profiling GI symptom patterns may improve symptom recognition and inform refinement of screening tools and future nutrition-related research [33]. Sex-related differences in physiology may influence GI function and symptom expression but remain largely absent from current assessments [13,18]. Given the absence of objective biomarkers and limited evidence, characterising GI symptom reporting following concussion in athletes represents an important first step toward informing future mechanistic and interventional research.

Therefore, this study aimed to describe the prevalence and patterns of GI symptoms reported among athletes with a history of concussion and to explore whether symptom prevalence and burden differed by sex and country of residence. We hypothesised that (i) >50% of athletes would report ≥1 GI symptom following concussion and that (ii) GI symptom burden would differ by sex.

## 2. Materials and Methods

This cross-sectional study was conducted in line with the Declaration of Helsinki (2013). Ethical approval was granted by the Atlantic Technological University Ethics Board for Cohort 1 (ATU-RSC_AC_15/12/2023) and by the Thomas Jefferson University Institutional Review Board for Cohort 2 (iRISID-2023-2443). Participants provided electronic informed consent prior to participation. Surveys were completed voluntarily and anonymously, and no personally identifiable information was recorded. All data were stored on encrypted, password-protected institutional cloud storage (OneDrive), with access restricted to authorised members of the research team. Data collection was conducted between January and August 2024 via Microsoft Forms (Version 2502; Microsoft Corporation, Redmond, WA, USA) for Cohort 1 and via Qualtrics (Qualtrics LLC, Provo, UT, USA) for Cohort 2.

### 2.1. Participants and Study Design

**Figure 1:**
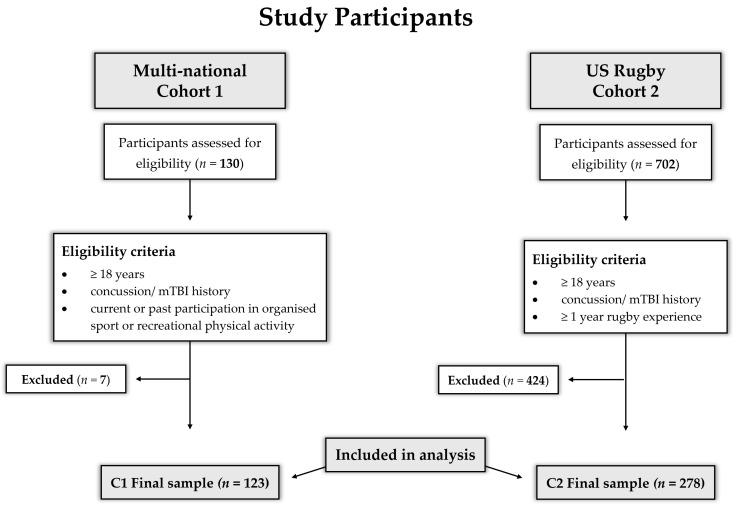
Flow diagram of participant recruitment, eligibility screening, exclusions, and final sample sizes for the multi-national cohort and the US rugby cohort in this observational study.

Two athlete cohorts were included for cross-national analysis (Figure 1): Cohort 1 comprised multi-national, multi-sport athletes (*n* = 123), and Cohort 2 comprised community rugby athletes (*n* = 278) from the United States (USA), hereafter referred to as US athletes.

#### 2.1.1. Cohort 1 (*n* = 123)

A total of 130 athletes were recruited using convenience sampling through email and social media platforms (Instagram, LinkedIn, X, Facebook), as well as sporting organisations, university departments, national players’ associations, club administrators, posters, and outreach to athletes and support personnel (e.g., coaches, nutritionists, physiotherapists). Seven participants (<18 years; 1 male, 6 females) were excluded, yielding a final analytical sample of 123 participants (94.6%).

#### 2.1.2. Cohort 2 (*n*= 278)

A total of 702 US community rugby participants were recruited through a national study on concussion behaviours, involving players, coaches, administrators, referees, and sports medicine staff. Of these, 424 did not meet the inclusion criteria (≥18 years, ≥1 year of full-contact rugby experience as a player, and history of rugby-related concussion), resulting in a final analytical sample of 278 participants (39.6%).

#### 2.1.3. Cohort Integration and Data Harmonisation

For cross-national comparisons, the three US participants (3/123) from Cohort 1 were combined with Cohort 2, yielding 281 US participants for pooled analyses. Core variables (demographics, concussion history, post-concussion GI symptoms) were harmonised across cohorts, retaining “country of residence” for stratified comparisons; GI symptom responses differed by cohort (Likert vs. binary yes/no format) and were harmonised to binary indicators of presence/absence for pooled analyses (Section 2.3). The recruitment and integration process is illustrated in Figure 1.

### 2.2. Survey Instruments and Measures

Structured surveys assessed demographics, sporting background (past and present), concussion history, and post-concussion symptomatology. Cohort 1 completed a 49-item survey that included self-reported dietary changes, supplement use, and access to professional care. Survey items referred to participants’ status at the time of survey completion (ToSC) or to experiences following their most recent concussion (Supplementary Table S1). These additional variables were not collected for Cohort 2 and were excluded from pooled analyses to ensure comparability. Information on dietary intake, alcohol consumption, medication use (including antibiotics and non-steroidal anti-inflammatory drugs [NSAIDs]), sleep quality, psychological stress, and training load was not systematically collected across cohorts. A definition of concussion and mTBI was provided to support reporting [1]. Both cohorts self-reported concussion history, including the number of concussions sustained during sport (general or rugby-specific for Cohort 2), recreational activities, or other settings. In Cohort 1, responses of ‘9+’ were coded as 10 for analysis (four athletes reported 10–13 events). Concussion totals also included two ≥50-event cases from Cohort 2 (totalling 100 and 39). Sensitivity analyses were conducted excluding ≥50-event cases. Additionally, exploratory multivariable logistic regression analyses including sex, country (restricted to the two largest groups: USA and Ireland), and lifetime concussion count were performed as robustness checks to assess the stability of findings.

#### Post-Concussion Symptom Assessment

Assessment instruments included GI-specific symptom items, the Rivermead Post-Concussion Symptoms Questionnaire (RPQ), and the SCAT-6 (Supplementary Tables S1–S3). As different instruments were administered across cohorts, Supplementary Table S4 summarises the symptom domains and instruments used prior to harmonisation. Analyses focused on 13 GI symptoms administered to both cohorts following their most recent concussion (Supplementary Table S5). To ensure comparability across cohorts, symptom responses were standardised prior to analysis. Cohort 1 completed the RPQ [34] and GI-specific symptoms using a 5-point Likert scale (0 = “not experienced” to 4 = “severe problem”), with ratings ≥ 1 indicating symptom presence. Cohort 2 assessed GI symptoms using binary (yes/no) responses. For pooled analyses, all responses were harmonised to binary indicators (presence/absence; see Section 2.3). Data quality assurance included duplicate checks and exclusion of incomplete responses.

General Post-Concussion Symptoms

General post-concussion symptoms were measured using two validated instruments: the RPQ [34,35] and the SCAT-6 symptom checklist [1], which assess somatic, emotional, and cognitive domains (Supplementary Tables S2 and S3). The RPQ comprises 16 symptoms rated on a 5-point Likert scale (0 = “not experienced”, 4 = “severe problem”), with total scores ranging from 0–64; ratings of 1 (“no more of a problem than before injury”) were coded as symptom presence to capture all post-injury experiences. The SCAT-6 includes 22 symptoms rated on a 7-point scale (0 = “none”, 6 = “severe”), with total scores ranging from 0–132 [1].

GI-Specific Symptoms

GI-specific symptoms were adapted from validated instruments: the Functional Gastrointestinal Disorders (FGD) Symptom Questionnaire (King’s College London and Guy’s & St Thomas’ NHS Foundation Trust) and the Gastrointestinal Symptom Rating Scale (GSRS) [36,37,38]. Thirteen symptoms common to both cohorts were analysed (Supplementary Tables S4 and S5), including nausea and/or vomiting (hereafter, nausea/vomiting), loss of/poor appetite, diarrhoea, abdominal pain/discomfort, increased flatulence/wind, indigestion/reflux/heartburn, new food sensitivities, dry skin/psoriasis/eczema, mouth sores/ulcers, acne/rosacea, food cravings, gastritis, and stomach ulcers.

Several GI-specific items assessed exclusively in Cohort 1 were excluded due to non-overlap between cohorts, including increased tiredness, constipation, belching/burping, stomach gurgling, urgency to open bowels, and incomplete evacuation. GI symptom data were analysed as binary indicators (presence/absence) for pooled comparisons. Nausea/vomiting was the only GI-related symptom assessed by the RPQ and SCAT-6 (despite wording differences), and its prevalence and severity were examined by sex and country.

### 2.3. Statistical Analysis

All primary analyses were exploratory and unadjusted. Data were cleaned and analysed using Microsoft Excel (Version 2502; Microsoft Corporation, Redmond, WA, USA). No missing data were observed in either cohort.

Descriptive statistics summarised participant characteristics, concussion history, and symptom prevalence (general and GI-specific). Categorical variables were reported as frequencies and percentages. Continuous and ordinal variables were summarised as means ± standard deviation (SD), median, interquartile range (IQR), and range. Normality was assessed using skewness and kurtosis thresholds alongside visual inspection. Statistical significance was set at *p* < 0.05.

Sports participation was self-reported and categorised by contact level: high (e.g., rugby, Gaelic football, American football), moderate (e.g., basketball, field hockey, gymnastics), or non-contact (e.g., athletics, cycling, swimming) [39]. Although Cohort 2 comprised rugby athletes exclusively, most participants in both cohorts reported high-contact sports participation; therefore, cohorts were not treated as distinct concussion-risk strata.

Age differences between sexes were tested using independent-samples *t*-tests. Non-normal variables (e.g., concussion counts and GI symptoms) were compared using Mann–Whitney *U* tests. Effect sizes included Cohen’s *d* for parametric tests, rank-biserial correlations (*r*) for non-parametric tests, and absolute risk differences (*ARD*) and odds ratios (*OR*) for chi-square (*χ*^2^) tests.

RPQ and SCAT-6 ratings were standardised to a 5-level ordinal scale: 0 = “not experienced” (RPQ 0; SCAT 0), 1 = resolved (RPQ 1; “no more of a problem”), 2 = mild (RPQ 2; SCAT 1–2), 3 = moderate (RPQ 3; SCAT 3–4), and 4 = severe (RPQ 4; SCAT 5–6).

GI-specific symptoms (13 items) were coded as binary indicators (presence/absence). Total GI symptom burden (range: 0–13) was calculated as the sum of present symptoms and analysed both as a continuous variable and as a binary outcome (any symptom vs. none). Symptoms were grouped into four domains: somatic (7 items), cognitive (3 items), emotional (3 items), and sleep (1 item) for analysis and do not represent validated subscales. Domain scores were calculated by summing ordinal item ratings (0–4). Descriptive statistics included mean ± SD, median, IQR, range, and the proportion reporting ≥1 symptom per domain.

Sex differences in symptom prevalence were examined using *χ*^2^ tests (*df* = 1), with Fisher’s exact tests applied when expected cell counts were <5. Domain-level sex differences were assessed using Mann–Whitney *U* tests.

Country-level analyses were exploratory and unadjusted. Due to small sample sizes for the United Kingdom (UK), Australia, and New Zealand, these groups were analysed descriptively only. Kruskal–Wallis tests assessed overall variation in GI symptom burden across countries; post hoc comparisons were limited to adequately sized groups and were not considered confirmatory. Exploratory multivariable logistic regression analyses were conducted as robustness checks and were restricted to the two largest country groups (USA and Ireland; Supplementary Table S6). Spearman’s rank-order correlations examined associations between domain scores, total GI symptom burden, and concussion counts, with coefficients interpreted using standard thresholds.

## 3. Results

A total of 401 participants (56.1% male; mean age 32.4 ± 11.3 years) with a history of concussion were included. Participants were predominantly from the USA (70.1%, *n* = 281) and Ireland (24.7%, *n* = 99); participants from the UK (4.2%, *n* = 17), Australia (0.7%, *n* = 3), and New Zealand (0.2%, *n* = 1) were analysed descriptively only.

Female participants were significantly younger than males (29.3 ± 8.3 vs. 34.7 ± 12.7 years; *t* (399) = 4.88, *p* < 0.001, Cohen’s *d* = 0.49). Detailed demographic characteristics are presented in Table 1.

### 3.1. Concussion History

Participants reported a total of 1553 concussions, averaging 3.9 ± 6.5 per individual (median = 3, IQR [1–4], range 1–100). The distribution was highly right skewed, reflecting a small sub-group with very high lifetime concussion counts (95th percentile = 10).

Males (*n* = 225) reported 1073 concussions (mean 4.8 ± 8.4; median = 3; IQR [1–5]; range 1–100), while females (n = 176) reported 480 concussions (mean 2.7 ± 2.1; median = 2; IQR [1–3]; range 1–15). Males reported significantly more concussions than females (Mann–Whitney *U* = 16,221, *Z* = −3.09, *p* < 0.002), representing a small-to-moderate effect size (rank-biserial correlation *r* = 0.18, common-language effect size = 0.59).

Overall, 6.2% of participants reported ≥10 sport-related concussions, including 9.8% of males (22/225) and 1.7% of females (3/176). Most concussions were reported among athletes in the US (73.5%; median = 3; IQR [1–4]) and Ireland (18.2%; median = 2; IQR [1–4]). Sex distributions varied by country, with a male predominance in the US and a more balanced distribution in Ireland; analyses beyond the US and Ireland are presented descriptively only (Supplementary Table S7).

### 3.2. Sport Participation by Contact Level

The majority of participants (94.5%, *n* = 379) reported participation in at least one high-contact sport (males: 56.5%; females: 43.5%), followed by moderate-contact (4.2%, *n* = 17; males: 52.9%; females: 47.1%) and minimal/non-contact sports (1.2%, *n* = 5; males: 40.0%, females: 60.0%). Of all reported concussions, 1486 (95.7%) were sport-related, with males accounting for 1037 (69.8%) and females 449 (30.2%). Recreational activities (e.g., cycling, horse riding) accounted for 30 concussions (1.9%), while other mechanisms (e.g., accidental falls, officiating, or medical support roles) contributed 37 concussions (2.4%).

### 3.3. Gastrointestinal Symptom Presence Post-Concussion

A total of 249 athletes (62.1%) reported at least one GI symptom following concussion, comprising 133 of 225 males (59.1%) and 116 of 176 females (65.9%), corresponding to 864 observations of GI symptom presence across 3237 total assessments (26.7%). Symptom absence was more common among males (92/225, 40.9%) than females (60/176, 34.1%); however, this difference was not statistically significant (*χ*^2^ (1) = 1.94, *p* = 0.164; *φ* = 0.07). Overall, 152 participants (37.9%) reported no GI symptoms.

Among participants reporting GI symptoms (*n* = 249; 53.4% males, 46.6% females), a total of 887 concussions were reported (mean 3.6 ± 4.9; median = 2, IQR [1–4]). The mean age was 29.5 ± 9.4 years (*t* (247) = 2.50, *p* = 0.013, Cohen’s *d* = 0.32). Most symptomatic participants were from the USA (144/249, 57.8%) and Ireland (85/249, 34.1%), with smaller proportions from the UK (17/249, 6.8%) and Australia (3/249, 1.2%). Most concussion events occurred during sport (853/887, 92.5%). Among symptomatic participants, the majority were players (183/249, 73.5%) and competed in high-contact sports (228/249, 91.6%).

**Figure 2:**
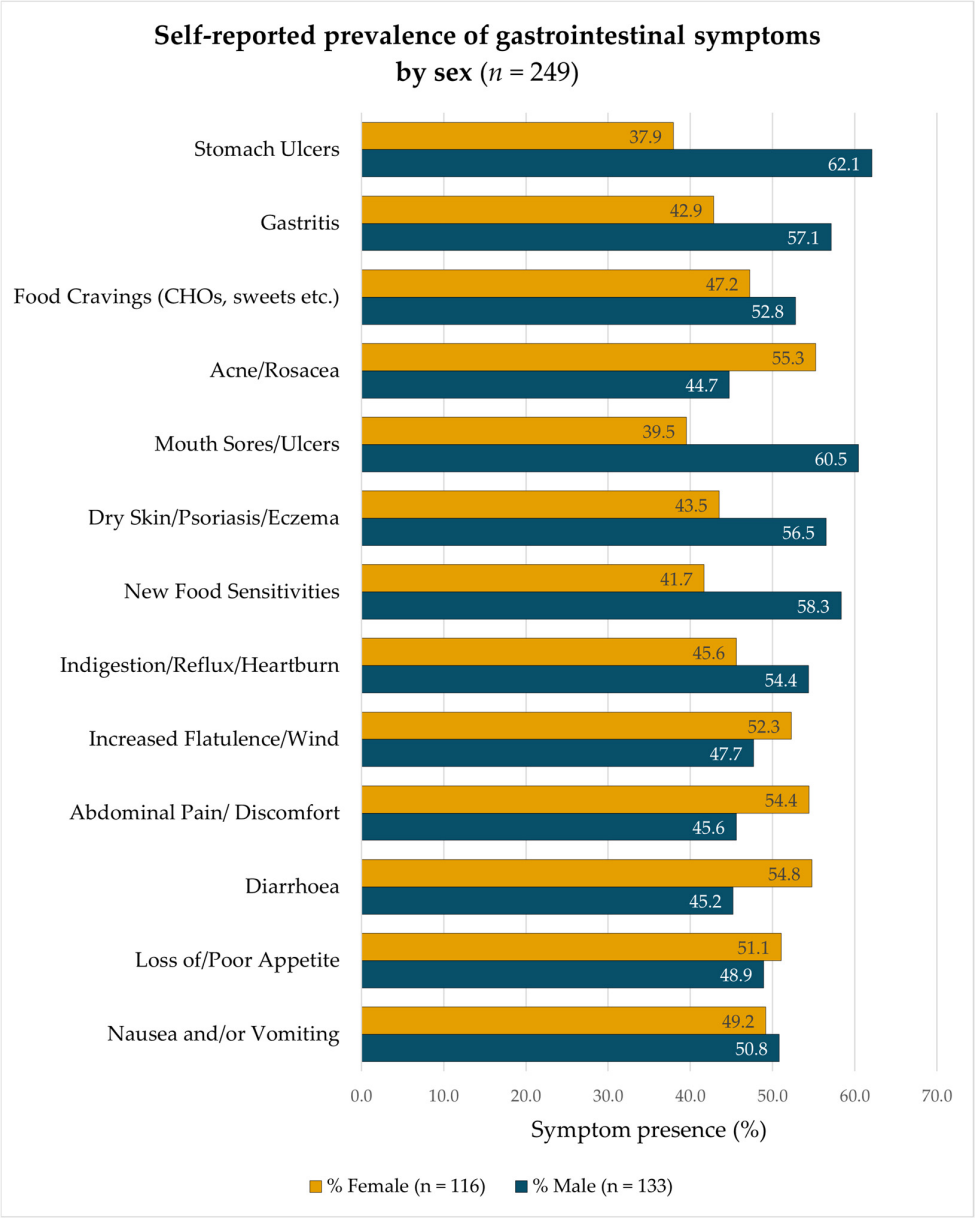
Self-reported prevalence of gastrointestinal (GI) symptoms by sex among athletes reporting at least one GI symptom (*n* = 249). Bars represent the percentage of male (*n* = 133) and female (*n* = 116) athletes reporting each symptom. Values are presented descriptively; inferential statistics are reported in Table 2. No statistically significant sex-based differences were identified.

Figure 2 illustrates the prevalence of individual GI symptoms by sex among athletes reporting at least one GI symptom.

**Table 2:** Prevalence of gastrointestinal (GI) symptom presence among athletes reporting at least one GI symptom (*n* = 249), by sex.

GI-Specific Symptom	Total, *N* (%) (*n* = 249)	Male, *N* (%) (*n* = 133)	Female, *N* (%) (*n* = 116)	ARD	OR	95%CI	χ^2^ (*df* = 1)	*p*
Nausea and/or Vomiting	185 (74.3)	94 (70.7)	91 (78.4)	+7.8	0.66	0.37–1.18	1.96	0.162
Loss of/Poor Appetite	141 (56.6)	69 (51.9)	72 (62.1)	+10.2	0.66	0.40–1.09	2.62	0.106
Diarrhoea	73 (29.3)	33 (24.8)	40 (34.5)	+9.7	0.63	0.36–1.09	2.80	0.094
Abdominal Pain/Discomfort	68 (27.3)	31 (23.3)	37 (31.9)	+8.6	0.65	0.37–1.14	2.30	0.129
Increased Flatulence/Wind	65 (26.1)	31 (23.3)	34 (29.3)	+6.0	0.73	0.42–1.29	1.16	0.282
Indigestion/Reflux/Heartburn	57 (22.9)	31 (23.3)	26 (22.4)	−0.9	1.05	0.58–1.90	0.03	0.867
New Food Sensitivities	48 (19.3)	28 (20.9)	20 (17.2)	−3.8	1.28	0.68–2.42	0.58	0.447
Dry Skin/Psoriasis/Eczema	46 (18.5)	26 (19.5)	20 (17.2)	−2.3	1.17	0.61–2.22	0.22	0.640
Mouth Sores/Ulcers	43 (17.3)	26 (19.5)	17 (14.7)	−4.9	1.42	0.72–2.76	1.04	0.308
Acne/Rosacea	38 (15.3)	17 (12.8)	21 (18.1)	+5.3	0.66	0.33–1.33	1.36	0.244
Food Cravings (CHOs, sweets, etc.)	36 (14.5)	19 (14.3)	17 (14.7)	+0.4	0.97	0.48–1.97	0.01	0.934
Gastritis	35 (14.1)	20 (15.0)	15 (12.9)	−2.1	1.19	0.58–2.45	0.23	0.633
Stomach Ulcers	29 (11.6)	18 (13.5)	11 (9.5)	−4.1	1.49	0.67–3.31	0.99	0.320
Total GI symptom observations	864	443 (51.3)	421 (48.7)					

Note: Data show the prevalence of each GI symptom among athletes reporting ≥1 GI symptom (*n* = 249), by sex. Percentages represent the proportion of each sex reporting each symptom. A total of 864 GI symptom-presence observations were recorded (443 in males [51.3%], 421 in females [48.7%]). Percentages represent the proportion of each sex reporting each symptom. Sex differences were assessed using chi-square tests chi-square tests (χ^2^, *df* = 1; *p* < 0.05). Effect sizes are presented as absolute risk differences (ARD; female% − male%) and odds ratios (OR) with 95% confidence intervals (CI). Post hoc power analyses (α = 0.05) indicated limited statistical power given the sample sizes (females *n* = 116; males *n* = 133), with, observed power across symptoms ranging from ~3–39%, and a minimum detectable ARD of ~9–17% at 80% power; therefore, non-significant findings may reflect limited power rather than absence of effect. GI = gastrointestinal; *N* = total sample; *n* = subgroup sample.

#### 3.3.1. Gastrointestinal Symptom Burden

Symptomatic participants reported a median of 2 GI symptoms (IQR [1–5]) and a mean of 3.5 ± 3.1 (range 1–13). Most experienced 1–5 symptoms (199/249, 79.9%), with 36 (14.5%) reporting 6–10 symptoms and 14 (5.6%) reporting ≥11 symptoms. Descriptively, symptom burden was broadly similar by sex: males reported a mean of 3.3 ± 3.1 (median = 2, IQR [1–4]) and females 3.6 ± 3.0 (median = 2, IQR [2–5]); in both groups, the majority reported 1–5 symptoms (males: 106/133, 82.0%; females: 90/116, 77.6%; Supplementary Table S8).

Nausea and/or vomiting was reported by nearly three-quarters of symptomatic athletes (*n* = 185, 74.3%), followed by loss of/poor appetite (*n* = 141, 56.6%). Other commonly reported symptoms included diarrhoea (*n* = 73, 29.3%), abdominal pain/discomfort (*n* = 68, 27.3%), and flatulence (*n* = 65, 26.1%). Less frequently reported symptoms (<25%) included indigestion/reflux/heartburn, new food sensitivities, dry skin/eczema, mouth sores/ulcers, acne/rosacea, food cravings, gastritis, and stomach ulcers. Complete GI symptom prevalence and sex-based comparisons are presented in Table 2.

#### 3.3.2. Sex-Based Differences in GI Symptom Reporting

Across 13 GI symptoms assessed, reporting patterns were broadly comparable between sexes, with negligible effects (*ARD* < 5%) for seven symptoms and small differences (*ARD* 5–20%) for six symptoms (Table 2). Females reported higher prevalence of nausea/vomiting (78.4% vs. 70.7%), loss of/poor appetite (62.1% vs. 51.9%), diarrhoea (34.5% vs. 24.8%), and abdominal pain/discomfort (31.9% vs. 23.3%) than males; however, none of these differences reached statistical significance (all *p* > 0.05). Males reported slightly higher prevalence of indigestion/reflux (23.3% vs. 22.4%), food sensitivities (20.9% vs. 17.2%), dry skin/psoriasis/eczema (19.5% vs. 17.2%), and stomach ulcers (13.5% vs. 9.5%); although males had higher odds of reporting ulcers (OR = 1.49), this difference was not statistically significant (Fisher’s exact *p* = 0.097; Table 2).

Total GI symptom counts did not differ by sex (Mann–Whitney *U* = 6975, *Z* = 1.30, *p* = 0.192). Given the sample sizes, power to detect small-to-moderate differences was limited (observed power ~3–39%); therefore, non-significant results should be interpreted cautiously as they reflect low power than true absence of effect.

#### 3.3.3. Country Differences in GI Symptom Reporting

GI symptom reporting varied by country. Prevalence was highest among Irish athletes (85/99, 85.9%) compared with US athletes (144/281, 51.2%). All athletes from the UK (17/17) and Australia (3/3) reported at least one GI symptom; however, these findings should be interpreted cautiously due to very small sample sizes.

Overall GI symptom burden differed across countries (Kruskal–Wallis *H* = 90.7, *df* = 3, *p* < 0.001). In exploratory post hoc analyses restricted to adequately sized groups, Irish athletes reported a higher GI symptom burden than US athletes (Bonferroni-adjusted Mann–Whitney *U* = 993, *Z* = −10.59, *p* < 0.001). Comparisons involving countries with very small sample sizes were considered descriptive only (Supplementary Table S9).

While most participants reported 1–5 symptoms, athletes from Ireland exhibited a broader distribution of GI symptom burden, with a higher proportion reporting 6–10 or ≥11 symptoms relative to US athletes. Across countries, nausea/vomiting, and loss of/poor appetite were the most frequently reported symptoms, with higher prevalence among Irish (78.8% and 76.5%) and UK athletes (76.5% and 70.6%) than among US athletes (71.5% and 43.8%). Flatulence was more common among Irish athletes (52.9%), whereas diarrhoea was more prevalent among US athletes (13.2%). Findings from the UK and Australia are presented descriptively only (Table S9). 

### 3.4. Post-Concussion Symptoms Reported (RPQ and SCAT)

All 401 participants completed the RPQ and SCAT, generating 3635 symptom reports across 14 items spanning somatic, cognitive, emotional, and sleep domains (Table 3). Symptom prevalence was high, with each item reported by more than 50% of participants. Severity ratings were predominantly mild (47.8%) to moderate (46.2%), with mean severity scores ranging from 1.3 to 2.1 and medians of 1–2; severe ratings were uncommon (6.0%; scores 5–6). The most frequently reported symptoms were fatigue/tiring easily and poor concentration (both 74.6%), followed by headaches (72.8%), irritability (69.8%), and sleep disturbance (67.6%).

Sex-stratified analyses showed no statistically significant differences for any RPQ/SCAT symptom or domain (all χ^2^ *p* > 0.05). Effect sizes were small (odds ratios ~1.0; absolute risk differences generally within ±7%). Although females showed slightly higher prevalence for some sensory and emotional symptoms (e.g., noise sensitivity, ARD = +6.9%), and for nausea/vomiting (ARD = +6.8%), these differences were not statistically significant. Notably, nausea/vomiting was the only GI-specific symptom captured by the RPQ/SCAT.

Overall, post-concussion symptoms were common but largely mild, dominated by somatic and cognitive complaints, with no evidence of sex-related differences in symptom reporting.

#### Nausea/Vomiting Severity Ratings by Sex

Across the full sample, 185 participants (46.1%) reported nausea/vomiting on the GI-specific item, while 206 (51.4%) endorsed nausea/vomiting on the RPQ/SCAT (Table 4). Reporting differed between the two measures: 29 (7.2%) participants reported nausea/vomiting only on the GI-specific item, 50 (12.5%) reported only on the RPQ/SCAT, and 156 (38.9%) reported nausea/vomiting on both measures. Among participants reporting nausea/vomiting on both assessments, severity was predominantly mild (46.8%) to moderate (44.9%), with only 8.3% rated as severe.

Country-level reporting patterns mirrored the overall trend. In the US (*n* = 281), 78 participants reported nausea/vomiting on both the GI-specific and RPQ/SCAT, 25 on the GI-specific only, and 35 on the RPQ/SCAT only. In Ireland (*n* = 99), 63 reported nausea/vomiting on both assessments, 4 on the GI-specific item only, and 12 on the RPQ/SCAT only. Smaller numbers observed in the UK (13 both, 1 RPQ only), Australia (2 both, 1 RPQ only), and New Zealand (no GI, but RPQ “2” rating).

Sex differences in nausea/vomiting reporting patterns were small. Females were slightly more likely to report nausea/vomiting on the GI-specific item only (10.2% vs. 4.9%; Cohen’s *h* = 0.21); however, severity was low overall (mean 1.3 ± 1.6; median of 1.0), and sex was not significantly associated reporting patterns (*χ^2^* (3, *N* = 401) = 6.28, *p* = 0.099, Cramer’s *V* = 0.13).

## 4. Discussion

This multi-cohort study indicates that GI symptoms were commonly reported among athletes with a history of concussion, with nearly two-thirds (62.1%) reporting at least one symptom. GI symptom profiles were broadly consistent across countries, and although symptoms were reported slightly more frequently by females than males (65.9% vs. 59.1%), sex-related differences were small and not statistically significant (all *p* > 0.05). Collectively, these findings suggest that post-concussion GI symptoms are prevalent across athlete populations and warrant consideration alongside other assessed symptom domains.

As the first study to examine post-concussion GI symptom burden across both sex and national cohorts, these findings demonstrate that such symptoms extend beyond those typically captured in standard concussion assessments. This suggests that GI symptoms may be underrepresented in current concussion research and assessment frameworks, with potential relevance for clinical assessment, recovery management, and return-to-play (RTP) decision-making. Together, these multi-cohort findings extend earlier Irish evidence [13], indicating that similar post-concussion GI symptom patterns may be observed across athletes from different countries and sporting contexts.

### 4.1. Concussion History and GI Symptom Prevalence

Concussion exposure was high in this cohort, with participants reporting a total of 1553 lifetime concussions (mean 3.9 ± 6.5 per athlete), predominantly sustained in sport and high-contact environments; approximately two-thirds of participants were current or recent athletes. Given the cross-sectional, self-reported design and wide variability in lifetime concussion exposure, temporal relationships and causality between concussion events and subsequent GI symptoms cannot be inferred.

GI symptoms were common, with 62.1% of athletes reporting at least one symptom (mean 3.5 ± 3.1). Most athletes reported 1–5 symptoms, with a smaller subset reporting ≥ 6, reflecting a broad symptom spectrum. The most prevalent symptoms were nausea/vomiting (74.3%), appetite loss (56.6%), diarrhoea (29.3%), abdominal pain/discomfort (27.3%), and flatulence (26.1%), which were consistent across cohorts.

These findings indicate that GI symptoms extend beyond nausea/vomiting, the only GI-related item in the SCAT-6 [1], and beyond the neurological, cognitive, emotional and sleep domains typically prioritised in concussion assessment tools [14,15]. These findings align with evidence describing appetite-related and digestive symptoms following concussion [13]; however, underlying mechanisms remain unclear and were beyond the scope of this study [24,25,26,40]. As appetite-related symptoms may influence dietary intake and fuelling behaviours during recovery, with downstream implications for energy availability and tissue repair, early recognition may support more comprehensive monitoring of post-concussion symptom burden [22,41].

#### 4.1.1. Sex Differences

Sex differences were minimal, with males and females reporting similar symptom counts (median = 2). Females reported slightly higher frequencies of nausea/vomiting, appetite loss, and abdominal pain, whereas males reported marginally more indigestion/reflux and stomach ulcers; none of these differences reached statistical significance [13,17]. Although hypothesised sex-linked influences on GBA communication, including autonomic and inflammatory processes described in the concussion literature, may contribute to GI symptom expression, these mechanisms were not assessed in the present study and require further investigation [26,42]. Hormonal fluctuations across the menstrual cycle may also contribute to variation in symptom expression, particularly among athletes of reproductive age [43,44].

Overall, the largely comparable GI symptom burden across sexes suggests that post-concussion GI symptom monitoring is relevant for athletes of all sexes, while supporting consideration of sex-sensitive monitoring within individualised concussion-recovery management.

#### 4.1.2. Country Differences

Country-level differences in GI symptom reporting were observed. Irish athletes reported a higher overall GI symptom burden than US athletes (*p* < 0.001) and a greater proportion reporting ≥6 symptoms. US athletes most commonly reported 1–5 symptoms, while findings from the UK and Australia should be interpreted cautiously due to small sample sizes. Symptom profiles were broadly similar across countries (nausea/vomiting and loss of/poor appetite most common), although flatulence was more frequent in Ireland and diarrhoea in the US. These variations may reflect cultural or contextual differences in symptom awareness, perception, and reporting behaviours; however, residual confounding cannot be excluded.

Together, these findings suggest that contextual factors may influence GI symptom reporting across athlete cohorts and support the inclusion of brief GI-specific screening alongside tools such as the SCAT-6 to support comprehensive symptom assessment and inform individualised rehabilitation planning.

### 4.2. Critical Gaps in Care: RPQ/SCAT vs. GI Limitations

Interpretation of these post-concussion findings warrants caution, as symptoms are multifactorial and non-specific and may pre-exist, co-occur with, or mimic concussion-related effects [1]. In athletes, GI symptoms are influenced by multiple non-concussion factors, including diet, supplement and alcohol use, psychological stress, sleep quality, training load, and medication use, particularly NSAIDs [45,46]. Psychological stress and sleep disturbances may also influence GI symptom expression via gut–brain mechanisms [47]. NSAIDs, commonly used to manage pain following sports injuries, are well documented to cause GI side effects such as nausea, early satiety, abdominal discomfort, diarrhoea, and gastritis [48,49]. Accordingly, these findings should be interpreted as reflecting symptom co-occurrence following concussion rather than causal attribution of GI symptoms to concussion.

Our findings demonstrate a critical gap in current concussion assessment practices. Concussion assessment tools such as the SCAT6 and RPQ capture only nausea/vomiting as a GI-related symptom [1,35], whereas athletes reported a broader spectrum of GI symptoms such as appetite loss, diarrhoea, flatulence, and abdominal pain. Discrepancies between GI-specific items and RPQ/SCAT responses were observed, with some participants endorsing symptoms on GI-specific items only, others on RPQ/SCAT only, and others on both with differing severity ratings [50,51].

These inconsistencies may reflect underreporting, delayed recognition, or differences in symptom interpretation. They also highlight limitations in current concussion assessment tools and support the inclusion of brief GI-specific items within standard concussion checklists. Although autonomic, inflammatory, and other GBA-related processes are hypothesised to influence GI symptom expression [26,42,51], these mechanisms were not assessed in the present study and remain an important area for further investigation.

### 4.3. Clinical Implications and Future Directions

GI-specific symptoms represent a prevalent but under-captured component of post-concussion symptomatology that is not consistently captured by standard assessment tools. Although overall symptom burden was generally low-to-moderate, the breadth of GI symptoms reported suggests that reliance on nausea/vomiting alone within tools such as the SCAT-6 and RPQ may underestimate athletes’ post-concussion symptom experiences [1,35]. While appetite-related symptoms were reported slightly more frequently in females, overall GI symptom burden did not differ significantly by sex, indicating that monitoring may be relevant across athlete populations.

Given this underrepresentation, incorporating brief GI-focused screening questions into post-concussion follow-up may support more comprehensive symptom assessment. As a pragmatic next step, a brief three-item GI screening protocol (assessing nausea/vomiting, appetite or food tolerance, and bowel-habit change) could be implemented following initial concussion assessments (e.g., SCAT-6) and evaluated for feasibility, acceptability, and construct validity in future studies. Such an approach may facilitate early identification of GI symptom burden without substantially increasing assessment load and inform the development of validated GI-specific screening tools for concussion care.

Where GI symptoms persist, referral to sports dietitians or nutritionists may support athletes with practical dietary strategies, including modifications to meal timing, smaller and more frequent meals, liquid-based options, and other supportive nutritional approaches [6]. GI symptoms may influence appetite, fuelling capacity, hydration status, and energy availability during recovery [52], with potential relevance to low energy availability (LEA) described in adolescents post-concussion [53]. These considerations highlight the value of nutrition-informed support within multidisciplinary, athlete-centred care pathways, although cross-national differences should be interpreted cautiously given the uneven sample sizes.

Emerging evidence implicates potential GBA processes relevant to GI symptom expression following concussion [24,25,26,42]; however, these mechanisms were not assessed in the present study and require further investigation. Future research should prioritise longitudinal designs incorporating validated GI-specific measures to clarify symptom trajectories, examine proposed GBA-related pathways, and evaluate the clinical utility of nutritional and supportive interventions. In addition, broader gut-health factors, including functional GI disorders and microbiota composition, warrant further exploration, and controlled trials with multidisciplinary follow-up may help inform best-practice management for athletes with persistent GI symptoms.

Collectively, these findings extend concussion research beyond neurological and cognitive domains by demonstrating that GI symptoms represent a prevalent and underrecognised component of post-concussion symptom experiences.

### 4.4. Limitations

This study has several limitations. First, the cross-sectional design precludes inference of causality or temporal relationships between concussion events and subsequent GI symptom reporting. Second, concussion history and GI-specific symptom reports were self-reported, introducing potential recall bias and misclassification risk, particularly regarding the number, timing, and severity of concussion events in the absence of clinical verification. Reported symptoms therefore reflect perceived experiences rather than clinically verified outcomes, limiting attribution to concussion when multiple influencing factors may be present. Time since most recent concussion was not available for Cohort 2 and was not included in analyses, restricting interpretation of symptom onset and duration.

Third, convenience sampling may limit generalisability and introduce self-selection bias, as athletes with greater concussion exposure or interest in concussion or GI symptoms may have been more likely to participate. Although multiple countries were represented, the sample was dominated by participants from the USA and Ireland, and cohort composition differed substantially: Cohort 1 comprised a multinational mixed-sport sample (*n* = 123) and Cohort 2 included only US community rugby players (*n* = 278). Consequently, country-level comparisons may be confounded by differences in sport type, contact exposure, medical access, concussion literacy, and small subsamples in some regions (e.g., UK, Australia, New Zealand), limiting interpretability.

Fourth, harmonisation of GI symptom data across cohorts was required due to use of Likert-scale responses in Cohort 1 and binary responses in Cohort 2. Dichotomisation to presence/absence indicators may have reduced sensitivity to symptom severity and limited detection of small effects. Although exploratory sensitivity analyses (including logistic regression) were conducted, fully adjusted multivariable modelling was constrained by limited statistical power (observed ~3–39%) and the absence of harmonised ordinal severity data and measured confounders across cohorts.

Finally, key confounders, including diet, hydration status, medication use (including NSAIDs), psychological stress, sleep quality, menstrual cycle phase, travel exposure, and pre-existing GI conditions, were not measured and may have influenced symptom reporting. Accordingly, post-concussion GI symptoms could not be differentiated from those attributable to unrelated or pre-existing GI conditions. Despite these limitations, the findings provide preliminary insight into an underrecognised symptom domain and highlight the need for standardised GI-specific measures and longitudinal research designs.

## 5. Conclusions

Gastrointestinal symptoms are a prevalent but underrepresented component of athletes’ self-reported symptom experiences following concussion. In this multi-cohort athlete sample, nearly two-thirds reported at least one GI symptom, highlighting a substantial symptom burden not captured by standard concussion assessment tools such as the SCAT-6 and RPQ. These findings extend existing concussion research beyond neurological and cognitive domains and suggest that GI symptoms may represent an overlooked aspect of athletes’ overall symptom experience.

Incorporating brief GI symptom screening into post-concussion assessment tools and frameworks may support improved recognition of GI symptom burden and contribute to more comprehensive, athlete-centred care. Further research should employ longitudinal designs with objective measures to validate GI-specific assessment tools, clarify symptom timing and trajectories following concussion, and evaluate targeted nutritional and clinical interventions to mitigate overall symptom burden.

## 6. Practical Applications

- Screen for GI symptoms routinely. Clinicians should monitor a broader spectrum of GI symptoms beyond nausea/vomiting following concussion, as these may influence recovery and readiness for RTP.
- Use multi-domain assessments. Evaluations should include GI, somatic, cognitive, emotional, sleep, and autonomic domains to avoid underestimating overall symptom burden.
- Consider contextual differences. Geographic and cultural factors may influence symptom awareness and reporting; clinicians should adapt assessment strategies accordingly.
- Integrate nutrition expertise. Collaboration with sports dietitians and nutritionists may support the management of appetite loss, hydration challenges, and energy deficits associated with GI symptoms following concussion.
- Refine concussion assessment tools. Incorporating GI-specific symptom items into the SCAT-6, RPQ, and future concussion assessment frameworks may improve symptom capture and clinical relevance.

## Figures and Tables

**Table 1:** Participant characteristics by sex, age, country of residence, sport participation, and concussion history (*N* = 401).

Variable	Total (*N* = 401)	Male	Female
Sex assigned at birth, *n* (%)	-	225 (56.1) ᵃ	176 (43.9)
Age (years), mean ± SD	32.4 ± 11.3	34.7 ± 12.7	29.3 ± 8.3
**Country**, *n* (%)			
Australia	3 (0.7)	3 (2.3)	-
Ireland	99 (24.7)	46 (46.5)	53 (53.5)
United Kingdom	17 (4.2)	11 (64.7)	6 (35.3)
New Zealand	1 (0.2)	1 (100.0)	-
United States ^b^	281 (70.1)	164 (58.4)	117 (41.6)
**Contact Sport**, *n* (%) ^c^			
High Contact	379 (94.5)	214 (56.5)	165 (43.5)
Moderate Contact	17 (4.2)	9 (52.9)	13 (47.1)
Non-to-Minimal	5 (1.2)	2 (40.0)	3 (60.0)
**Concussion History**			
Total reported, *n* (%) ^d^	1553	1073 (69.1)	480 (30.9)
Mean ± SD	3.9 ± 6.5	4.8 ± 8.4	2.7 ± 2.1
Median [IQR]	3 [1–4]	3 [1–5]	3 [1–4]

Note: *N* denotes the total sample size; *n* denotes subgroup counts. All values are presented as *n* (%) unless otherwise specified. Continuous variables are reported as mean ± SD or median [IQR]. SD = Standard Deviation; IQR = interquartile range. ᵃ Two participants identified as women and were included as female for sex-based analysis. ^b^ US participants (*n* = 281) include 3 from Cohort 1 (1 male [33.3%], 2 females [66.7%]) and 278 from Cohort 2 (163 males [58.6%], 115 females [41.4%]). ^c^ Contact sport category was based on the highest-contact sport reported; participants were classified as high contact if any high contact sport was listed, otherwise as moderate or non-to-minimal contact. ^d^ Concussion counts are reported as raw event totals; in Cohort 1, ‘9+’ responses were coded as 10 (four athletes reported 10–13 events). Totals include two ≥50-event outliers from Cohort 2 (100 and 39 events).

**Table 3:** Comparison of RPQ and SCAT-6 concussion symptoms reporting by sex (*N* = 401).

Domain	Concussion Symptom	Symptom Present, *n* (%)	Mild [1–2]	Moderate [3–4]	Severe [5–6]	Mean ± SD	Median [IQR]	Min–Max	95% CI
Somatic	Fatigue, tired more easily	299 (74.6)	117	166	16	2.1 (±1.6)	2.0 [3]	0–6	0.16
	Headaches	292 (72.8)	108	168	16	2.0 (±1.6)	2.0 [3]	0–6	0.15
	Noise sensitivity	251 (62.6)	129	112	10	1.5 (±1.5)	1.0 [3]	0–6	0.15
	Light sensitivity	247 (61.6)	116	119	12	1.6 (±1.6)	1.0 [3]	0–6	0.15
	Dizziness	245 (61.1)	100	122	23	1.7 (±1.7)	2.0 [3]	0–6	0.16
	Blurred vision (unfocused)	215 (53.6)	121	85	9	1.3 (±1.5)	1.0 [2]	0–6	0.14
	Nausea and/or vomiting *	206 (51.4)	104	84	18	1.3 (±1.6)	1.0 [3]	0–6	0.16
Cognitive	Poor concentration *	299 (74.6)	142	135	22	2.0 (±1.6)	2.0 [3]	0–6	0.16
	Forgetful/poor memory	270 (67.3)	130	123	17	1.8 (±1.6)	1.0 [3]	0–6	0.15
	Slowed thinking	253 (63.1)	119	124	10	1.7 (±1.6)	1.5 [3]	0–6	0.16
Emotional	Irritability	280 (69.8)	136	119	25	1.8 (±1.6)	1.0 [3]	0–6	0.16
	Sadness/depression	260 (64.8)	156	89	15	1.6 (±1.6)	1.0 [3]	0–6	0.15
	Emotional/frustration	247 (61.6)	111	125	11	1.6 (±1.6)	1.0 [3]	0–6	0.16
Sleep	Sleep disturbance/ trouble falling asleep	271 (67.6)	148	108	15	1.7 (±1.5)	1.0 [3]	0–6	0.15

Note: A total of 3635 symptom reports were recorded (1613 from females, 44.4%, and 2022 from males 55.6%). Percentages indicate the proportion of the total sample reporting each symptom. Where severity is presented, percentages reflect the proportion of symptomatic participants at each severity level. * Denotes gastrointestinal (GI)-specific symptoms.

**Table 4:** Participant severity ratings of nausea and/or vomiting by sex (*N* = 401).

Nausea/Vomiting Severity	None (0)	Mild (1–2)	Moderate (3–4)	Severe (5–6)	Total (*n*)	%
**All participants**	195	104	84	18	401	100.0
- GI-specific item	29	73	70	13	185	46.1
**Female (*n* = 176)**	84	41	44	7	176	43.9
- GI-specific item	18	32	35	6	91	22.7
**Male (*n* = 225)**	111	63	40	11	225	56.1
- GI-specific item	11	41	35	7	94	23.4
**Sex differences in nausea/vomiting reporting across symptom items (*N* = 401).**						
**Reporting Category**	**Total (*N* = 401)**		**Male (*n* = 225)**		**Female (*n* = 176)**	
Present–GI-specific & RPQ	156 (38.9)		83 (36.9)		83 (36.9)	
Present–GI-specific only	29 (7.2)		11 (4.9)		11 (4.9)	
Present–RPQ only	50 (12.5)		31 (13.8)		31 (13.8)	
Absent–both	166 (41.4)		100 (44.4)		100 (44.4)	
Total	401 (100.0)		225 (56.1)		225 (56.1)	

Note: Values represent the number of participants who rated nausea/vomiting severity on RPQ /SCAT and GI-specific items. Percentages are based on the total sample (*N* = 401). Chi-square test results: χ^2^ (3) = 6.28, *p* = 0.099; Cramer’s *V* = 0.13 (small effect).

## Data Availability

The original data are unavailable to protect participant confidentiality. However, the data presented in this article are included in the Supplementary Materials. Further inquiries can be directed to the corresponding author.

## Supplementary Materials

The following supporting information can be downloaded at https://www.mdpi.com/article/10.3390/nu18111740/s1. Table S1: Gastrointestinal symptomology assessment administered to Cohort 1 participants (*N* = 130). Table S2: Post-concussion symptom assessment (RPQ) administered to Cohort 1 participants (*N* = 130). Table S3: Gastrointestinal symptomology assessment administered to Cohort 2 participants. Table S4: Summary of symptom instruments assessed in Cohort 1 and Cohort 2 prior to harmonisation. Table S5: Gastrointestinal symptom items administered to Cohort 1 and 2 participants. Table S6: Exploratory multivariable logistic regression examining factors associated with reporting ≥1 GI symptom. Table S7: Self-reported concussion history by country and sex (descriptive; *N* = 401). Table S8: Grouped distribution of GI symptom presence and burden among symptomatic participants (*n* = 249) by sex. Table S9: Cross-national gastrointestinal (GI) symptom distribution by country and sex (*n* = 249; ≥1 GI symptom).

## Abbreviations

The following abbreviations are used in this manuscript:

ARD	Absolute risk difference
CISG	Concussion in Sport Group
FGD	Functional Gastrointestinal Disorders Symptom Questionnaire
GBA	Gut–brain axis
GCS	Glasgow Coma Scale
GI	Gastrointestinal
GSRS	Gastrointestinal Symptom Rating Scale
IQR	Interquartile range
LEA	Low energy availability
mTBI	Mild traumatic brain injury
OR	Odds ratio
RPQ	Rivermead Post-Concussion Symptoms Questionnaire
RTP	Return to play
SCAT	Sport Concussion Assessment Tool
SD	Standard deviation
ToSC	Time of survey completion
US	United States
UK	United Kingdom
USA	United States of America
U	Mann–Whitney U
χ^2^	Chi-square test
Z	Z score
