# Supplementary material for: Gastrointestinal Symptoms After Sport-Related Concussion: Prevalence and Patterns in a Multi-Cohort Analysis
Source: Nutrients. 2026 May 29;18(11):1740. doi: 10.3390/nu18111740 (PMC13258330; doi:10.3390/nu18111740)
Supplement: Supplementary file 1 [file nutrients-18-01740-s001.zip › nutrients-4267514-supplementary.pdf]

Gastrointestinal Symptoms After Sport-Related Concussion: Prevalence and Patterns in a Multi-Cohort Analysis

Emma Finnegan [1], Ed Daly [1], Katherine J. Hunzinger [2,3] and Lisa Ryan [1,*]

Supplementary Methods

To improve transparency regarding measurement equivalence across cohorts, Supplementary Table S5 summarises the symptom instruments and domains assessed in Cohort 1 and Cohort 2 prior to harmonisation. Detailed symptom lists and harmonisation procedures are provided in Supplementary Tables S1–S4. GI-specific and general post-concussion symptom assessments administered to Cohort 1 are shown in Supplementary Tables S1 and S2, while those administered to Cohort 2 are shown in Supplementary Table S3.

Cohort 1 – *Multi-national Athletes*

Table S1. Gastrointestinal symptom assessment administered to *Cohort 1* participants (*N* = 130).

**Definition**	“A concussion or mild traumatic brain injury (mTBI) are head injuries that occur following a direct or indirect impact to the head, face, neck, or other body parts that passes an impulsive force to the brain. This force disrupts brain function, leading to the rapid onset of transient symptoms. [1]”	
**Item No.**	**Survey Item**	**Response Format**
**7**	Have you previously experienced a concussion/head injury?	Yes/No
**38**	Please review the gastrointestinal (GI) symptoms or conditions below. Reflect on your experience after your concussion/ mTBI/ head injury impact/ diagnosis and indicate each of them occurred after your most concussion/mTBI.<br>**Symptom** list rated:<br>1. Loss of appetite,<br>2. Nausea and/or Vomiting,<br>3. Diarrhoea,<br>4. Constipation,<br>5. Stomach ulcers,<br>6. Gastritis,<br>7. New food sensitivities,<br>8. Mouth sores/ulcers,<br>9. Indigestion/heartburn,<br>10. Incomplete evacuation, Dry skin/eczema,<br>11. Acne/rosacea,<br>12. Food cravings,<br>13. Abdominal pain,<br>14. Bloating,<br>15. Flatulence,<br>16. Belching,<br>17. Gurgling,<br>18. Urgency to open bowels,<br>19. Increased tiredness	Likert scale: 0 = Not experienced; 1 = No more of a problem; 2 = Mild problem; 3 = Moderate problem; 4 = Severe problem.
39	Did you experience any other GI-type difficulties?	Free text
40	Rate any additional symptoms listed in Q39.	Likert scale

Note: This section of the survey was administered only to Cohort 1 participants (*n* = 130). Responses were used to assess post-concussion gastrointestinal (GI) symptom prevalence and severity. GI symptoms were rated using a 5-point Likert scale. The 20 GI-specific items were adapted from the Functional Gastrointestinal Disorders (FGD) Symptom Questionnaire and the Gastrointestinal Symptom Rating Scale (GSRS) [36–38]. The “acute phase” was defined as 24–72 hours and up to 7 days following the most recent concussion or mTBI event [10].

Table S2. Post-concussion symptom assessment (RPQ) administered to Cohort 1 participants ($N = 130$).

**Definition**	"A concussion or mild traumatic brain injury (mTBI) are head injuries that occur following a direct or indirect impact to the head, face, neck, or other body parts that passes an impulsive force to the brain. This force disrupts brain function, leading to the rapid onset of transient symptoms." [1]	
**Item No.**	**Survey Item**	**Response Format**
32	Rivermead Post-Concussion Symptoms Questionnaire (RPQ).<br>Adapted from King et al. (1995) [34].<br><br>Did you experience any of the following symptoms or conditions within the initial 24 hours following your concussion/ mTBI/ head injury impact and/or diagnosis?<br>**Symptoms** rated individually:<br>1. Headaches<br>2. Feelings of dizziness<br>3. Nausea and/or vomiting<br>4. Noise sensitivity, easily upset by loud noise<br>5. Sleep disturbance<br>6. Fatigue, tiring more easily<br>7. Being irritable, easily angered<br>8. Feeling depressed or tearful<br>9. Feeling frustrated or impatient<br>10. Poor memory, feeling forgetful<br>11. Poor concentration<br>12. Taking longer to think<br>13. Blurred vision (unfocused, fuzzy)<br>14. Light sensitivity or easily upset by bright light<br>15. Double vision<br>16. Restlessness	Likert scale:<br>0 = Not experienced; 1 = No more of a problem; 2 = Mild problem; 3 = Moderate problem; 4 = Severe problem.
33, 35	Did you experience any other difficulties?<br>Rate symptoms listed (Q33 and Q35)	Free text; Likert scale (above)

Note: This section of the survey was administered only to Cohort 1 participants ($n = 130$). Responses were used to assess post-concussion symptom prevalence and severity. Symptoms were rated using a 5-point Likert scale. The Rivermead Post-Concussion Symptoms Questionnaire (RPQ) [34] was used to assess acute post-concussion or mTBI symptom severity across emotional, cognitive, and neurological domains. For this study, the "acute phase" was defined as 24–72 hours and up to 7 days following the most recent concussion or mTBI event [10].

Cohort 2 – *U.S. Rugby Participants*

Table S3. Gastrointestinal symptom assessment administered to *Cohort 2* participants.

<table>
<tr><th>Section</th><th>Survey Item</th><th>Response Format</th></tr>
<tr><td>GI-specific symptom items</td><td>Please review the gastrointestinal (GI) symptoms or conditions listed below. Did you experience any of the following symptoms after your most recent rugby-related concussion?
GI-related symptoms listed
1. Loss of/poor appetite
2. Nausea and/or Vomiting
3. Diarrhea
4. Stomach Ulcers
5. Gastritis
6. New food intolerances
7. Mouth sores/Ulcers
8. Indigestion/Heartburn
9. Flatulence and gas
10. Abdominal pain and bloating
11. Dry skin/psoriasis or eczema
12. Acne/rosacea
13. Cravings for sweets/carbohydrates
14. None of the above</td><td>Binary (Yes/No)</td></tr>
<tr><td>SCAT-6 symptom checklist [1] items</td><td>Please rate each of the following symptoms based on how you typically felt after your most recent rugby-related concussion.
Symptoms rated:
1. Headaches
2. Pressure in head
3. Neck pain
4. Nausea or vomiting
5. Dizziness
6. Blurred vision
7. Balance problems
8. Sensitivity to light
9. Sensitivity to noise
10. Feeling slowed down
11. Feeling like “in a fog”
12. “Don’t feel right”
13. Difficulty concentrating
14. Difficulty remembering
15. Fatigue or low energy
16. Confusion
17. Drowsiness
18. More emotional
19. Irritability
20. Sadness
21. Nervous or anxious
22. Trouble falling asleep (if applicable)</td><td>Likert scale: 0 = Not experienced; 1 = No more of a problem; 2 = Mild problem; 3 = Moderate problem; 4 = Severe problem.</td></tr>
</table>

Note: This section of the survey was administered only to Cohort 2 participants ($n = 279$). Responses were used to assess post-concussion gastrointestinal (GI) symptom prevalence. GI symptoms were reported using a binary (yes/no) response format without severity ratings, while general post-concussion symptom severity was assessed separately using the SCAT-6 symptom checklist [1]. The 13 GI-specific items were adapted from validated instruments, including the Functional Gastrointestinal Disorders (FGD) Symptom Questionnaire and the Gastrointestinal Symptom Rating Scale (GSRS) [36–38]. Participants could select multiple symptoms.

Harmonised GI Symptoms Used in Pooled Analyses

Table S4. Gastrointestinal symptom items administered to *Cohort 1 and Cohort 2* participants.

Section	Survey Item Description	Response Format
**GI-specific symptom** items	Please review the gastrointestinal (GI) symptoms listed below. Did you experience any of the following symptoms after your recent **concussion or mTBI** (including rugby-related)?<br>**GI-related symptoms** listed<br>1. Nausea and/or Vomiting<br>2. Loss of/ Poor Appetite<br>3. Diarrhoea<br>4. Abdominal Pain/ Discomfort<br>5. Increased Flatulence/ Wind/ Gas<br>6. Indigestion/ Reflux/ Heartburn<br>7. New Food Intolerances /Sensitivities<br>8. Dry Skin/ Psoriasis/ Eczema<br>9. Mouth Sores/ Ulcers<br>10. Acne/ Rosacea<br>11. Food Cravings (CHOs, sweets etc.)<br>12. Gastritis<br>13. Stomach Ulcers	**Cohort 1: Likert scale** **(**0 – 4)<br><br>**Cohort 2: Binary** (0 = No, 1 = Yes)<br><br>**Harmonised indicators:** Presence/absence

Note: For pooled analyses, 13 GI symptom items common to both cohorts were harmonised to binary indicators (presence vs. absence). In Cohort 1, items were originally rated on a 0–4 Likert scale, with any rating ≥ 1 coded as symptom present. In Cohort 2, items were collected directly using binary (yes/no) responses.

Overview of Symptom Instruments and Harmonisation

Table S5. Summary of symptom instruments assessed in Cohort 1 and Cohort 2 prior to harmonisation.

Symptom domain	Instrument	Cohort 1	Cohort 2	Response format
GI-specific symptoms	GI-specific symptoms: 13 items (FGD/GSRS-derived)	✓	✓	Likert (Cohort 1) / Yes–No (Cohort 2)
	Additional GI-specific symptoms (7 items; Cohort 1 only)	✓	X	Likert
	"None of the above" item	X	✓	Yes–No
General post-concussion symptoms	RPQ	✓	X	Likert
	SCAT-6	X	✓	Likert
Nausea/vomiting	GI-specific symptom (FGD/GSRS-derived)	✓	✓	Yes–No
	RPQ	✓	X	Likert
	SCAT-6	X	✓	Likert

Note: For pooled analyses, 13 GI-specific symptoms common to both cohorts were harmonised to binary indicators (presence/absence). In Cohort 1, items were originally rated on a 0–4 Likert scale; in Cohort 2, items were collected using binary (yes/no) responses. ✓ = assessed; X = not assessed. Detailed symptom lists and harmonisation measures are provided in Supplementary Tables S1–S4.

Exploratory Sensitivity Analysis

Exploratory sensitivity analyses were conducted to assess the robustness of associations between GI symptom presence and key participant characteristics. Analyses were restricted to the two largest country groups (USA and Ireland) for model stability, and additional sensitivity checks excluded participants reporting extreme lifetime concussion counts (≥50 events).

Table S6. Exploratory multivariable logistic regression examining factors associated with reporting ≥1 GI symptom.

Predictor	Odds Ratio	95% CI	*p*-value
**Sex** (female vs male)	1.22	0.78–1.90	0.385
**Country** (Ireland vs USA)	5.56	3.01–10.28	<0.001
**Concussion count** (per event)	0.98	0.95–1.02	0.374

Note: Odds ratios (OR) are presented from an exploratory multivariable logistic regression assessing GI symptom presence (present/absent), with sex, country (Ireland vs USA), and lifetime concussion count entered simultaneously as predictors. The USA served as the reference category. Analyses were restricted to the USA and Ireland to improve model stability and are reported for robustness purposes only.

Supplementary Results

Table S7. Self-reported concussion history by country and sex (descriptive; *N* = 401).

Variable	Australia	Ireland	United Kingdom	New Zealand	United States
Participants, *n* (%)	3 (0.7)	99 (24.7)	17 (4.2)	1 (0.2)	281 (70.1)
Male, *n* (%)	3 (100.0)	46 (46.5)	11 (64.7)	1 (100.0)	164 (58.4)
Female, *n* (%)	–	53 (53.5)	6 (35.3)	–	117 (41.6)
Total concussions self-reported, *n* (%)	23 (1.5)	282 (18.2)	105 (6.8)	2 (0.1)	1141 (73.5)
Median per participant	10 (range 5–11)	2 [1–4]	4 [2–6]	2	3 [1–4]

Note: Values are presented as *n* (%) unless otherwise stated. Percentages for males and females are calculated within each country sample (total *N* = 401; 225 males, 176 females). The overall sample comprised 401 participants, including 225 males and 176 females. Concussion counts are reported as raw event totals; in Cohort 1, responses of '9+' were coded as 10 (four athletes reported 10–13 events). Totals include two ≥50-event outliers from Cohort 2 (100 and 39 events). Medians are reported due to skewed distributions; IQRs are shown where sample size permits, ranges are reported for Australia (*n* = 3), and median only for New Zealand (*n* = 1).

Table S8. Grouped distribution of GI symptom presence and burden among symptomatic participants (*n* = 249) by sex.

Symptom Range	Total, *n* (%)	Male, *n* (%)	Female, *n* (%)
**Overall burden**	249	133 (53.4)	116 (46.9)
Mean ± SD, Median [IQR]	3.5 ± 3.1, 2.0 [1–5]	3.3 ± 3.1, 2.0 [1–4]	3.6 ± 3.0, 2.0 [2–5]
1–5	199 (79.9)	109 (82.0)	90 (77.6)
6–10	36 (14.5)	16 (12.0)	20 (17.2)
11–13	14 (5.6)	8 (6.0)	6 (5.2)

Note. Values are presented as *n* (%) unless otherwise stated. Percentages within male and female columns are calculated relative to that sex's sample. SD = Standard Deviation; IQR = interquartile range; *n* = sample size; *N* = total sample.

Table S9. Cross-national gastrointestinal (GI) symptom distribution by country and sex (n = 249; ≥1 GI symptom).

**Country,** ***n*** (%)	**Symptom Count**	**Total,** ***n*** (%)	**Male,** ***n*** (%)	**Female,** ***n*** (%)
**USA, 114** (57.8)	1–5	139 (96.5)	77 (96.3)	62 (96.9)
	6–10	4 (2.8)	3 (3.8)	1 (1.6)
	11–13	1 (0.7)	-	1 (1.6)
**Ireland, 85** (34.1)	1–5	49 (57.6)	25 (64.1)	24 (52.2)
	6–10	26 (30.6)	9 (23.1)	17 (37.0)
	11–13	10 (11.8)	5 (12.8)	5 (10.9)
**UK, 17** (*6.8*)	1–5	8 (47.1)	4 (36.4)	6 (66.7)
	6–10	6 (35.3)	4 (36.4)	2 (33.3)
	11–13	3 (17.6)	3 (27.3)	-
**Australia, 3** (*1.2*)	**1–5**	3 (1.2)	3 (100.0)	-

Note: Percentages in the Country, *n* (%) column represents the proportion of athletes within the symptomatic sample ($n = 249$). Percentages for symptom ranges are calculated within each country subgroup. Data for the UK and Australia are presented descriptively only due to very small sample sizes. Overall group differences were assessed using Kruskal–Wallis tests ($H = 90.7$, $df = 3$, $p < 0.001$); post-hoc comparisons were restricted to adequately sized groups and are reported narratively in the Results. GI = gastrointestinal; *n* = subgroup sample.
